# Potassium channel KCN11 is required for maintaining cellular osmolarity during nitrogen starvation to control proper cell physiology and TAG accumulation in *Chlamydomonas reinhardtii*

**DOI:** 10.1186/s13068-020-01769-x

**Published:** 2020-07-20

**Authors:** Feifei Xu, Junmin Pan

**Affiliations:** 1 MOE Key Laboratory of Protein Sciences, School of Life Sciences, Tsinghua University, Beijing, China; 2 Department of Anesthesiology and Perioperative Medicine, Xijing Hospital, The Fourth Military Medical University, Xi’an, Shaanxi China; 3 Laboratory for Marine Biology and Biotechnology, Qingdao National Laboratory for Marine Science and Technology, Qingdao, Shandong China

**Keywords:** *Chlamydomonas*, Osmoregulation, KCN11, N starvation, TAG

## Abstract

**Background:**

Nitrogen (N) starvation in algae induces a variety of structural and metabolic changes including accumulation of triacylglycerol (TAG). Given the promising prospect of using algae as feedstock for biofuel production, accumulation of TAG upon N starvation becomes an ideal system to study TAG biosynthesis. Under nitrogen-depleted conditions, algae also accumulate compatible solutes such as sugar and certain amino acids, which is expected to elevate osmolarity in the cytoplasm. However, how osmoregulation is maintained and how it impacts on carbon metabolism, especially TAG accumulation under N starvation, are not well understood.

**Results:**

We show here that potassium channel KCN11 localized in the contractile vacuole (CV) mediates osmoregulation during N starvation and loss of KCN11 profoundly affects cell physiology and TAG biosynthesis. KCN11 level is increased and the CV pulsation is accelerated. Loss of KCN11 induces aberrant CV cycle, inhibition of cell growth, increase of cell size, inhibition of chlorophyll loss and TAG accumulation. These effects are rescued by addition of sucrose to raise osmolarity in the culture medium, indicating that osmoregulation is required for cell adaptation to N starvation. Metabolomic analysis shows reduction of acetyl-CoA and accumulation of glyceraldehyde-3-phosphate in *kcn11* mutant relative to the control under N starvation, indicating that defects in acetyl-CoA biosynthesis and some metabolic steps from glyceraldehyde-3-phosphate to TAG contribute to the decreased TAG accumulation due to loss of osmoregulation.

**Conclusions:**

This work provides novel insight of osmoregulation during N starvation in the control of cell physiology and metabolism especially TAG accumulation. According to these findings, we propose that osmolarity should be carefully monitored during the industrial production of biodiesel.

## Background

Nitrogen (N) is a key element for the growth and development of plant cells and algae. The impact of N deficiency on gene expression and metabolism in *Chlamydomonas reinhardtii*, a widely used model for studying various fundamental processes, has been studied for decades. This includes studies of photosynthesis and of the activation of the gametogenesis program [1–4]. These studies documented the changes in significant physiological reprogramming, such as ribosome abundance, starch accumulation, and organization of the thylakoid membrane [5–9]. Interest in these pathways has renewed recently because of the use of *C. reinhardtii* as a reference organism for understanding triacylglycerol (TAG) accumulation pathways induced by N starvation [10–16].

Recently, a transcriptomic analysis in *Neochloris oleoabundans* has reported that N starvation leads to a significant change in the metabolism of both sugar and proline; in particular, the conversion from UDP-glucose to sucrose is strongly enhanced under nitrogen-depleted condition [17]. Sugars and proline are main groups of compatible solutes found in algae [18]. In seed plants, N starvation also induces accumulation of amino acids that function as compatible solutes [19]. Synthesis and degradation of compatible solutes (also known as “low molecular weight metabolites”) are important mechanisms for achieving osmotic balance. Osmotic adjustment is pivotal for cell survival and metabolism in unicellular algae. The regulatory responses of algae include changes of cell volume, intracellular ion concentration, intracellular glycerol concentration, and the expression of some genes [20, 21]. Inhibited growth and photosynthetic rates by osmotic stress are also observed in *Chlamydomonas reinhardtii* [22]. Under N starvation conditions in *Chlamydomonas*, it is unknown whether osmoregulation is changed relative to normal growth conditions, and how osmoregulation is maintained. Furthermore, how osmoregulation affects TAG accumulation is not clear.

The osmoregulation of *Chlamydomonas reinhardii* is mediated by contractile vacuoles (CVs). Two CVs, localized in the cell anterior near the flagellar base, pulsate alternatively to remove excessive water from the cytoplasm to maintain cellular osmolarity [23, 24]. Our previous study showed that a potassium channel KCN11 is located in the membrane of the contractive vacuole. Loss of KCN11 in a *kcn11* mutant generated by DNA insertional mutagenesis disrupts proper pulsation of the contractile vacuole and osmoregulation [25], suggesting that KCN11 is important for osmoregulation. It has been reported that the expression of *KCN11* was up-regulated during N starvation [12]. Thus, we aim to investigate the role of KCN11 in osmoregulation as well as metabolic changes under N starvation.

## Results

### Elevation of KCN11 level and contractile vacuole cycling rate during N starvation

**Fig. 1:**
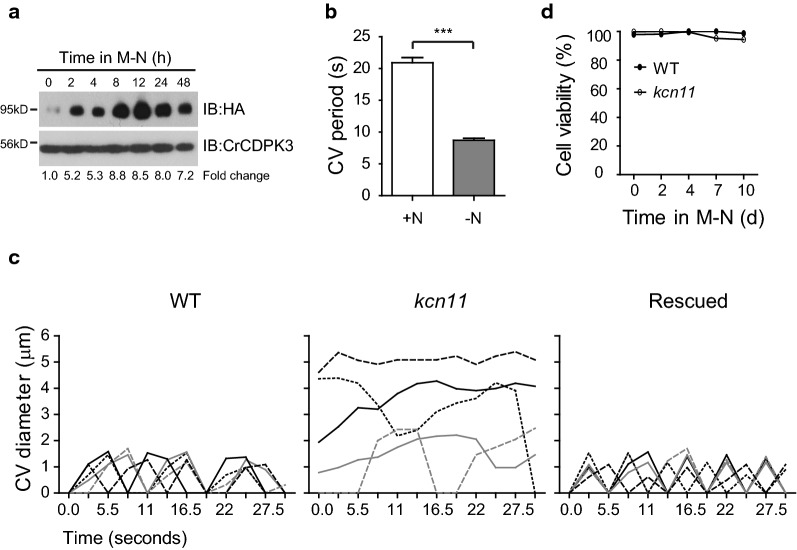
KCN11 is required for CV pulsation during N starvation. **a** The level of KCN11 is elevated upon N starvation. Cells expressing *KCN11*-*HA* were transferred from N-replete (time 0) to N-depleted medium followed by immunoblotting. CrCDPK3 was used as a loading control. **b** Faster CV pulsation occurs in N-depleted medium. Wild-type cells grown in N-replete (+N) medium or starved for nitrogen (−N) for 24 h were measured for the CV periods. Data shown are mean ± sem (*n* = 30 cells). ***P < 0.0001 (*t* test). In the following experiments, cells were starved for nitrogen. **c** Diagram showing the CV cycles of the wild-type cells, *kcn11* and rescued cells after N starvation for 24 h. Each line represents the CV cycle from individual CV. **d** Cell viability grown in N-starved medium. Cells were stained by FDA. At least 300 cells were scored from each time point. Data shown are mean ± sem from three independent experiments

Transcriptional analysis showed that the expression of *KCN11* mRNA is strongly induced upon N starvation [12]. To determine whether the protein level of KCN11 is also increased, *kcn11* rescued cells expressing HA-tagged *KCN11* were transferred from N-replete to N-depleted media followed by immunoblot analysis. As shown in Fig. 1a, the level of KCN11 was increased several folds shortly after cells being transferred to N-delete medium and remained highly expressed for a period of 48 h. Next, we examined CV cycling, a process that the CV absorbs water from the cytoplasm followed by water discharge to the outside of the cell repeatedly, which is an indicator of osmoregulation [25]. Compared to N-replete conditions, the contractile vacuole period under N-depleted conditions was decreased approximately by half, which reflects an increase of CV cycling rate (Fig. 1b). These data suggest that N starvation increases cellular osmolarity, which is accompanied with increased protein level of KCN11 and CV cycling rate.

### The *kcn11* mutant fails to expel liquid from the cells efficiently

Our previous work showed that under normal growth condition, KCN11 does not affect the CV cycle. However, under hypotonic condition, CV pulsation is accelerated and lack of *KCN11* impaired CV pulsation and cell growth [25]. The increase of cellular osmolarity during N starvation would increase water influx. Such a situation is similar to a hypotonic condition. To determine whether KCN11 is required for CV pulsation during N starvation, the CV cycles were measured. In wild-type cells and rescued cells, the CVs pulsate with an oscillating pattern (Fig. 1c). In contrast, the CV cycles of N-starved *kcn11* were too aberrant to do the quantitative analysis. Some CVs did not or slowly pulsate with abnormal large CV size (Fig. 1c). These data demonstrate that KCN11 is essential for CV pulsation during N starvation. Nevertheless, the *kcn11* mutant is able to survive in the absence of nitrogen (Fig. 1d), indicating that osmoregulation is not essential for cell survival under N deprivation.

### Loss of KCN11 alters cell morphology, growth and chlorophyll content

**Fig. 2:**
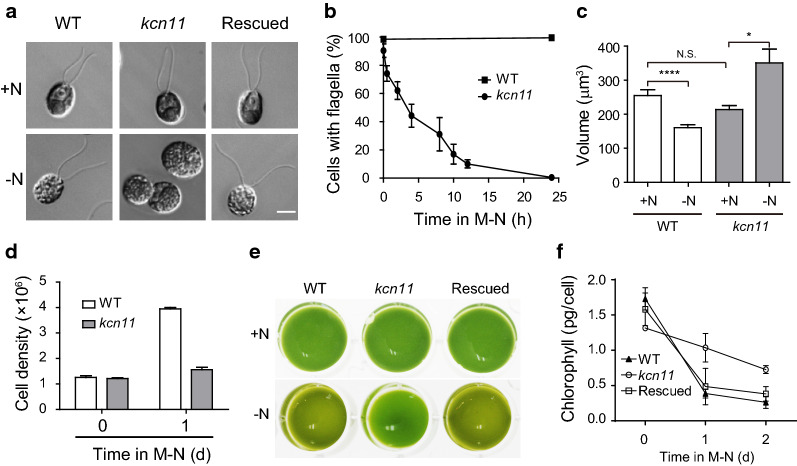
KCN11 deletion results in abnormal cell morphology and cell growth. **a**, **b** Flagella loss caused by *KCN11* mutation under N-depleted condition. Shown are representative cell images after N starvation for 24 h (**a**) and flagellar loss kinetics during N starvation (**b**). Data shown are mean ± sem (*n* = 200). Bar = 5 μm. **c** Cell volume increase in *kcn11* under N-depleted condition. Cells were measured after 24 h at the indicated media. Data shown are mean ± sem (*n* = 200). N.S., no significant. ***P < 0.0001 (*t* test). **d** Cell growth of *kcn11* was inhibited following N starvation. Data shown are mean ± sem (*n* = 200). **e**, **f** Loss of chlorophyll is retarded in *kcn11* following N starvation. Images of cell cultures grown in N-replete or N-depleted conditions for 24 h (**e**). Chlorophyll content during N starvation (**f**). Data shown are mean ± sem (*n* = 3)

Loss of KCN11 impairs CV pulsation during N starvation, which is expected to affect osmoregulation leading to defects in various cellular processes. Microscopic examination found that *kcn11* mutant cells gradually lost their flagella during N starvation (Fig. 2a, b). The cell size decreased during N starvation for wild-type cells. In contrast, the cell size was increased in *kcn11* mutant cells (Fig. 2c). Within 1 day of N starvation, wild-type cells were still able to divide. However, cell proliferation in the *kcn11* mutant was inhibited (Fig. 2d). We also found that KCN11 affected chlorophyll content of the cell. The cellular level of chlorophyll decreased rapidly in wild-type cells during a 2-day culture in N-deficient conditions (from 1.73 to 0.26 pg per cell). In contrast, the cellular level of chlorophyll in *kcn11* cells decreased much slower (from 1.32 to 0.73 pg per cell) (Fig. 2e, f).

### KCN11 affects TAG accumulation during N starvation

**Fig. 3:**
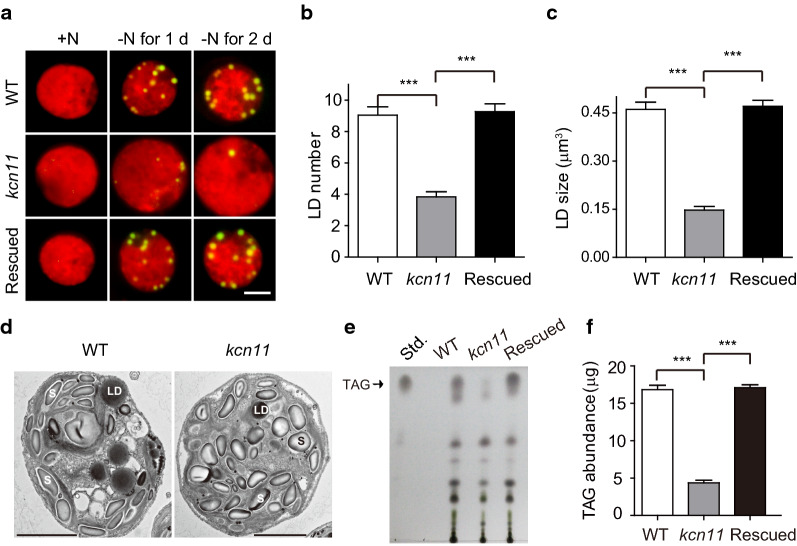
KCN11 affects TAG accumulation. **a** Representative images of cells stained with Nile Red. Red: chlorophyll autofluorescence; Yellow: lipid droplets. Bar = 5 μm. **b**, **c** Statistical analysis of the number (**b**) and size (**c**) of the lipid droplets. Cells were starved for nitrogen for 2 days. Data shown are mean ± sem (*n* = 50). ***P < 0.0001 (*t* test). **d** Transmission electron microscopy images of WT and *kcn11* cells after 2 days of nitrogen depletion. S: starch granule; LD: lipid droplet. Bar = 2 µm. **e**, **f** TAG analysis by thin-layer chromatography. Shown are representative image (**e**) and quantification (**f**). Cells were starved for nitrogen for 2 days. Data shown are mean ± sem (*n* = 3). ***P < 0.0001 (*t* test)

Accumulation of TAG is induced during N starvation in *Chlamydomonas* [25]. We then evaluated the influence of KCN11 on the accumulation of lipid. In contrast to wild-type and rescued cells, *kcn11* mutant cells barely formed lipid droplets shown by Nile Red staining (Fig. 3a). The cellular number and size of the lipid droplets were greatly reduced in the *kcn11* mutant relative to the control cells (Fig. 3b, c). These observations were consistent with analysis by electronic microscopy (Fig. 3d). We analyzed TAG amount by TLC (Fig. 3e, f). The amount of TAG in *kcn11* mutant was 4.35 µg per 4 × 10^6^ cells, which is 25.89% of that in wild-type cells (16.8 µg per 4 × 10^6^ cells) after 2 days of N deficiency. These data demonstrate that KCN11 is required for TAG accumulation during N starvation.

### Relieving osmotic stress and/or potassium stress rescues defects caused by KCN11 deletion

**Fig. 4:**
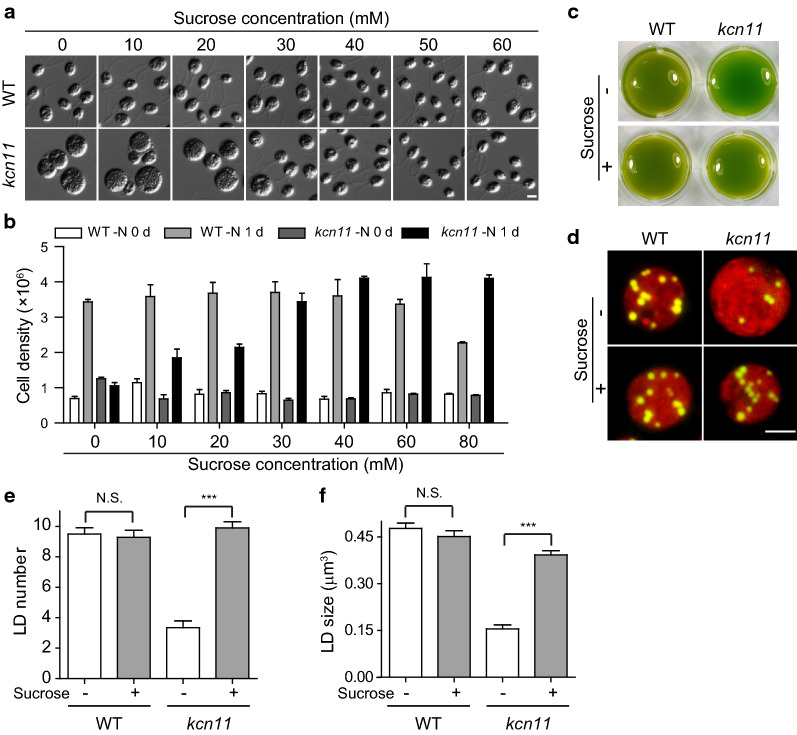
Rescue of *kcn11* phenotypes by elevation of osmolarity via addition of sucrose. **a** WT or *kcn11* cells were treated with different concentrations of sucrose for 1 day after depletion of nitrogen. Bar = 5 μm. **b** Cell growth of WT or *kcn11* cells in N-depleted media for 1 day with addition of different concentrations of sucrose. **c** WT or *kcn11* cells were grown in N-depleted media for 2 days in the presence or absence of 40 mM sucrose. **d**–**f** WT and *kcn11* cells were grown in N-depleted media for 2 days with or without 40 mM sucrose followed by Nile Red staining (**d**), by measuring cellular number (**e**) and size (**f**) of the lipid droplets. Bar = 5 µm. Data shown are mean ± sem (*n* = 50). N.S., no significant. ***P < 0.0001 (*t* test)

The defect in CV cycle in *kcn11* mutant cells suggests that loss of osmoregulation may be responsible for various defective cellular processes mentioned above during N starvation. It remains to be demonstrated experimentally that this is indeed the case. To this end, we elevated the osmolarity of the N-depleted culture medium by adding different concentrations of sucrose. With increasing concentrations of sucrose, we found that the cell size of the *kcn11* mutant gradually decreased and approached normal cell size at 40 mM sucrose (Fig. 4a). We also observed that cell growth of the *kcn11* mutant was also rescued at concentrations above 40 mM sucrose (Fig. 4b). Next, we examined the effect of 40 mM sucrose on the cellular chlorophyll level and TAG accumulation. Adding sucrose barely affected the chlorophyll content of the wild-type cells; however, that of the *kcn11* mutant decreased (Fig. 4c). Microscopic analysis showed that addition of sucrose also rescued the number and size of the droplets in *kcn11* mutant cells (Fig. 4d–f).

**Fig. 5:**
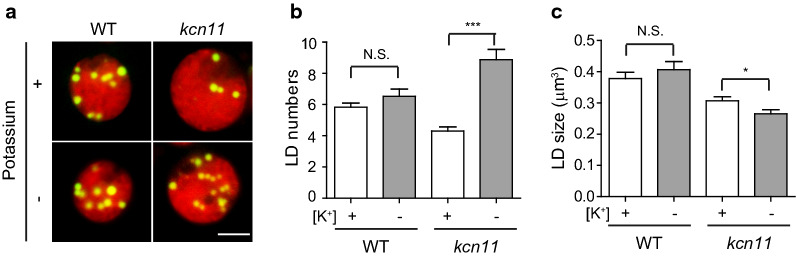
Removing potassium from culture media partially rescues formation of lipid droplets. WT or *kcn11* cells were grown in N-depleted medium for 2 days with or without potassium followed by Nile Red staining (**a**), by measurement of cellular number (**b**) and size (**c**) of the lipid droplets. Bar = 5 µm. Data shown are mean ± sem (*n* = 50). N.S., no significant. *P < 0.05, ***P < 0.0001 (*t* test)

Considering KCN11 is an outwardly potassium channel localized in the CV membrane [25], disruption of KCN11 may lead to an increase of potassium concentration in the cytoplasm. Abnormal accumulation of potassium in the cytoplasm may also contribute to some of the defective phenotypes in the *kcn11* mutant. Potassium was replaced with sodium in the N-depleted medium. The cell size, growth and chlorophyll content of *kcn11* mutant cells were not rescued (data not shown). Interestingly, the number of lipid droplets was apparently increased in the *kcn11* mutant when potassium was omitted (Fig. 5a, b). However, the size of the lipid droplets was not rescued (Fig. 5a, c). These data suggest that osmoregulation impaired by the absence of KCN11 is required for various aspects of cell physiology and metabolism following N deprivation.

### Analysis of carbon metabolism affected by loss of KCN11

**Fig. 6:**
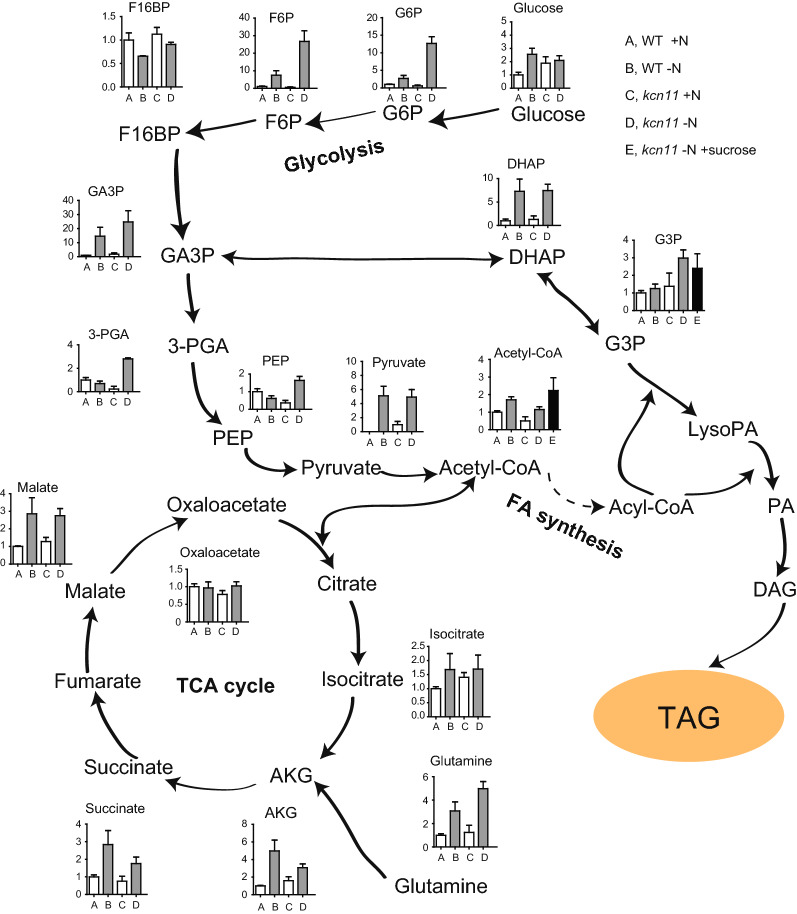
Metabolic analysis of metabolites in the glycolytic pathway and the Krebs cycle in WT and *kcn11* cells that were grown in N-depleted media for 2 days, respectively. G3P and acetyl-CoA were also assayed in *kcn11* cells supplemented with 40 mM sucrose. G6P: Glucose-6-phosphate; F6P: fructose-6-phosphate; F16BP: fructose-1,6-diphosphate; GA3P: glyceraldehyde-3-phosphate; 3-PGA: 3-Phosphoglyceric acid; DHAP: Dihydroxyacetone phosphate; PEP: phosphoenolpyruvic acid; G3P: glycerol phosphate; LysoPA: lysophosphatide; PA: phosphatidic acid; DAG: diacylglycerol; TAG: triacylglycerol; AKG: Alpha-ketoglutarate

Glycolysis and TCA cycle provide the raw materials and reducing power for TAG de novo synthesis. In view of this, we investigated the main metabolites in the glycolysis pathway and the TCA cycle in N-replete and N-depleted media of wild-type and *kcn11* cells, respectively (Fig. 6). This analysis may reveal the metabolic basis for TAG accumulation during N starvation and the metabolic steps for TAG accumulation that are profoundly affected by failure of osmoregulation due to loss of KCN11.

Compared to nitrogen-replete condition, a lot of metabolites in the glycolytic pathway during N starvation such as glucose-6-phosphate (G6P), fructose-6-phosphate (F6P), glyceraldehyde-3-phosphate (GA3P) and dihydroxyacetone phosphate (DHAP) and pyruvate increased significantly during nitrogen deficiency, both for wild-type and *kcn11* cells. Please note that DHAP is an important substrate for lipid synthesis and acetyl-CoA provided by pyruvate is a “building block” for fatty acids’ synthesis. The glycolytic metabolites (especially DHAP and pyruvate) may account for the production of fatty acids and lipids during nitrogen deficiency. Likewise, compared to nitrogen-replete condition, a lot of metabolites in the Krebs cycle such as isocitrate, α-ketoglutarate (AKG), succinate and malate increased significantly during nitrogen deficiency, which is also consistent for TAG accumulation under this condition. We also observed that the levels of some metabolites were decreased under N-depleted conditions. The reasons are unknown. It may reflect that some of the metabolites have higher turnover rates.

Acetyl-CoA and G3P are the end-products derived from the glycolytic pathway and/or the TCA cycle for TAG biosynthesis. The level of acetyl-CoA was decreased in the N-depleted *kcn11* mutant relative to N-depleted wild-type cells. This may suggest that the lower level of acetyl-CoA may contribute to the inhibition of TAG synthesis in *kcn11* mutant during N starvation. Interestingly, the level of G3P is much higher in the N-depleted *kcn11* mutant relative to N-depleted wild-type cells. Given that *kcn11* mutant was unable to accumulate TAG, it suggests that some of the downstream metabolic steps toward TAG synthesis may be defective in *kcn11* mutant during N starvation. To determine whether these effects were caused by failed osmoregulation due to *KCN11* mutation, we measured the levels of acetyl-CoA and G3P in N-delete *kcn11* cells in the presence of 40 mM sucrose. As expected, the level of acetyl-CoA was increased while that of G3P was decreased relative to the control without addition of sucrose. Thus, we propose that osmoregulation mediated by KCN11 affects the production of acetyl-CoA and some metabolic steps from G3P to TAG.

## Discussion

We have discovered that osmoregulation mediated by KCN11 plays a critical role in cell physiology and metabolism during N starvation in *C. reinhardtii*. We showed that the level of KCN11 was elevated and the rate of CV pulsation was increased when cells were cultured in N-depleted medium. Deletion of KCN11 in the *kcn11* mutant resulted in impairment of the CV cycle. These data demonstrate that N starvation increases cellular osmolarity, which is counter-balanced by accelerated CV pulsation. What is the trigger for inducing such a change in CV pulsation? One hypothesis is that the increased osmolarity resulting from accumulation of small metabolites in the cytosol would allow more water to flow in from outside the cell due to osmotic pressure. The CV would be filled up quickly with water from the cytoplasm, which is subsequently discharged to the outside, thus increased CV cycling is entailed. The level of KCN11 is elevated under nitrogen deficiency, it is interesting to explore whether there is a correlation between KCN11 level and CV period that reflects cytosolic osmolarity by analyzing cells at different times after onset of N depletion. Under N starvation, except for defects in CV pulsation, *kcn11* is also defective in regulation of cell growth and size and metabolic changes such as chlorophyll loss and TAG accumulation. KCN11 is a potassium channel; disruption of KCN11 may also result in elevation of cellular potassium, which may cause potassium toxicity leading to physiological defects [26]. However, omitting potassium can only partially restore TAG accumulation but other defects. In contrast, raising the osmolarity in the culture medium by addition of sucrose rescues all the defects, indicating that osmoregulation is pivotal for proper cell physiology and metabolism.

As an important molecule that is present at the intersection of multiple biochemical pathways, acetyl-CoA can be produced by several metabolic pathways, including glycolysis, TCA cycle, amino acid catabolism and lipids’ oxidation. TAG synthesis requires acetyl-CoA as well as G3P. Our metabolic study shows lots of upstream glycolytic metabolites in *kcn11* mutant were more than those in wild type following N deprivation, but there was no significant difference in pyruvate content and even less acetyl-CoA in *kcn11* mutant, indicating that enzymes of initial glycolytic pathway which are primarily in the chloroplast were activated, while pyruvate kinase which is primarily cytosolic and pyruvate dehydrogenase which is primarily in the chloroplast and mitochondria were suppressed in the *kcn11* mutant [27, 28]. We have also observed there is more glutamine in *kcn11*, suggesting a block from glutamine to acetyl-CoA, which probably occurs in the mitochondria [27]. G3P is backbone of TAG and starting material for TAG synthesis. The higher level of G3P in *kcn11* under N-depleted conditions suggests that G3P may not be efficiently utilized and accumulated. It indicates that some of the metabolic steps downstream of G3P which is primarily in the chloroplast are negatively affected [27]. Studies in diatom showed that carbon limitation by low light reorients carbon metabolism to production of phosphoenolpyruvic acid (PEP) and/or pyruvate, indicating that these metabolites are critical for carbon and downstream metabolisms [29, 30]. The suppression of production of pyruvate and its immediate product acetyl-CoA in the *kcn11* mutant are consistent with their critical role in lipid biosynthesis. In addition, our findings are consistent with previous studies that alteration of osmolarity could induce changes in protein conformational equilibria and modulate the activities of enzymes [31, 32].

Microalgae accumulate TAG as energy storage during N starvation. As TAG can be turned into biofuel after transesterification with methanol, algae have been regarded as one of the most promising feedstocks for the production of biofuels. A major limitation is that nitrogen deprivation provokes several side effects such as impairments in photosynthesis [33], as well as the slow down and cessation of cell growth [34]. For a long time, researchers have tried a variety of methods to realize the mutual improvement of both biomass and TAG [35–38]; however, the outcome is barely satisfactory [39]. Several studies showed that manipulation of intracellular carbon flow can promote the synthesis of TAG in cells. For example, *sta6* mutant is able to accumulate more TAG because of the lack of small subunit of ADPGlc pyrophosphorylase for starch formation [40]. Indeed, a comparative study has shown that several metabolic processes are altered in the *sta6* mutant; therefore, detailed analysis toward carbon flux in mutants affecting TAG synthesis may contribute to the understanding of the relationship between carbon metabolism and TAG synthesis [41]. So far, only a few mutants that are defective in TAG accumulation have been found in *Chlamydomonas*. For example, starch-accumulating mutants (e.g., *sta6 [ADP*-*glucose pyrophosphorylase], sta7*-*10 [isoamylase], bgal1 [β*-*galactosidase*-*like protein]*), acyl-editing mutants (e.g., *pgd1 [plastid galactoglycerolipid degradation 1], tgd2 [trigalactosyldiacylglycerol 2], pdat1 [phospholipid:diacylglycerol acyltransferase 1]*) and TAG mobilization mutants (e.g., *lip4 [lipase 4*]) [16, 38, 42–45]. The *kcn11* mutant is a new addition to this mutant library. Other mutants of CV function may show similar phenotypes [23]. In addition, considering the important role of osmotic stress in carbon metabolism [22], the current study provides a new perspective for the investigation of TAG accumulation mechanism.

## Conclusions

Loss of KCN11, a protein located in the membrane of the contractile vacuole (CV), profoundly affects cell physiology and TAG biosynthesis under N starvation. These effects are rescued by raising osmolarity in the N-depleted culture medium. Further metabolomic analysis reveals that the metabolic basis for TAG accumulation and the metabolic steps for TAG accumulation are affected by failure of osmoregulation. These results indicate an important role of osmolarity in controlling proper cell physiology and TAG accumulation during N deprivation. According to our findings, we propose that osmolarity should be carefully monitored during the industrial production of biodiesel.

## Materials and methods

### Stains, culture conditions and morphological analysis

*Chlamydomonas reinhardtii* strain 21gr (*mt*^+^) (*CC*-*1690*) is available from the *Chlamydomonas* Genetics Center, University of Minnesota. Generation of a *kcn11* mutant and a rescued strain expressing *KCN11*-*HA* was as described previously [25]. Cells were cultured in 250-ml erlenmeyer flasks with aeration with normal air at 23 °C under a 14 h-light–10 h-dark cycle. The light intensity was ~ 2000 Lux. For studies of N deficiency, cells grown in M medium were centrifuged, washed and further cultured in M–N medium at continuous illumination. Cell densities were estimated by counting fixed cells (1% glutaraldehyde) in the blood counting chamber. A Zeiss Axio Observer Z1 microscope (Zeiss) equipped with an electron microscope charge-coupled device camera (QuantEM512SC, Photometrics) was applied for cell imaging. Cell volumes were calculated based on the ellipsoid shape of *Chlamydomonas* cells using the formula 4/3*π*[*L*/2][*W*/2]^2^ [46], where *L* is cell length and *W* cell width, both of them were measured using ImageJ software (National Institutes of Health).

### Live-cell imaging

Contractile vacuole cycle was measured as described previously using two microscopy imaging systems [25]. The microscopy system described above was used to take images every 50 ms for statistical analysis and a Zeiss LSM780 META Observer Z1 Confocal Laser Microscope with 100× objective (NA1.40) was used to present the CV cycle for each cell.

Cells were stained with FDA (20 µg/ml, final concentration) for 5 min in the dark at room temperature and the samples were captured under Zeiss Axio Observer Z1 microscope equipped with a CCD camera (QuantEM 512SC, Photometrics) using a 40× objective to calculate the cell survival rate.

### Measurement of chlorophyll content and lipid analysis

Chlorophyll of cell pellet was extracted using 95% (v/v) ethanol and chlorophyll concentrations were calculated after measuring absorbance at 663 and 645 nm. Total chlorophyll was determined using equation, [Chl] (mg/l) = 20.2 * OD645 + 8.02 * OD663 [47]. For neutral lipids staining, cells starved for different time as indicated were stained with Nile Red (1 μg/ml in DMSO, final concentration, Sigma) for 5 min in the dark. Images were acquired using the Zeiss microscope (see above) equipped with 100 × oil objective lens. The Nile Red and chlorophyll signals were captured using the green (BP, 515–565 nm) and red (BP, 575–640 nm) fluorescence filters (Zeiss, Germany), respectively. Total lipids were extracted in a chloroform:methanol (1:2, v/v) system. After centrifuged at 3000*×g* for 10 min, the lower organic phase was transferred into a glass tube, dried at 70 °C under N_2_ gas and dissolved again in dimethylbenzene. The protein phase between water and organic phase was dried and dissolved in 0.2 M KOH at 37 °C overnight, the concentration determined by BSA assay was used as loading control. Neutral lipids (TAG) were separated by thin-layer chromatography (TLC) using silica plates developed with *n*-hexane:diethyl ether:acetic acid (70:30:1, v/v) and visualized by spraying the plates with CuSO_4_–H_3_PO_4_ (10% CuSO_4_ w/v, 10% H_3_PO_4_ v/v) followed by heating at 120 °C. ImageJ software was used for the quantification (https://imagej.nih.gov/ij/) (NIH, USA).

### SDS-PAGE and immunoblotting

SDS-PAGE and immunoblotting analysis were essentially as described previously [48]. Primary antibodies used are rat monoclonal anti-HA (1:2000, clone 3F10, Roche) and rabbit anti-CrCDPK3 antibody [49].

### Metabolomic analysis

Metabolites were simply extracted in a water–methanol–chloroform mixture (1:2:1, v:v:v) after metabolic activity quenching by injecting cells into 70% methanol (1:5, v:v). Targeted metabolomic experiment was analyzed by TSQ Quantiva. C18-based reverse-phase chromatography was utilized with 10 mM tributylamine, 15 mM acetate in water and 100% methanol as mobile phase A and B, respectively. This analysis focused on TCA cycle, glycolysis pathway, pentose phosphate pathway, amino acids and purine metabolism. In this experiment, we used a 25-min gradient from 5 to 90% mobile B. Positive–negative ion switching mode was performed for data acquisition. Cycle time was set as 1 s and a total of 300 ion pairs were included. The resolutions for Q1 and Q3 are both 0.7 FWHM. The source parameters are as follows: 3500 v for positive and 2500 v for negative ion mode; capillary temperature: 320 °C; heater temperature: 300 °C; sheath gas flow rate: 35; auxiliary gas flow rate: 10. Data analysis and quantitation were performed by the software Tracefinder (Thermo Fisher, CA).

The Dionex Ultimate 3000 UPLC system was coupled to a Q Exactive orbitrap mass spectrometer (Thermo Fisher, CA), equipped with a heated electrospray ionization (HESI) probe in positive ion mode. Extracts were separated by an ACQUITY UPLC HSS T3 column (2.1 × 100 mm, 1.8 μm, Waters). A binary solvent system was used, in which mobile phase A consisted of 5 mM ammonium acetate and 100% H_2_O, and mobile phase B of 100% methanol. A 10-min gradient with flow rate of 250 μl/min was used as follows: 0–2 min at 1% B; 2–4 min, 1–30% B; 4–6 min, 30–98% B; 6–7.1 min, 98% B; and 7.1–10 min, 1% B. Column chamber and sample tray were held at 35 °C and 10 °C, respectively. The data with mass range m/z 700–1500 were acquired by data-dependent MSMS acquisition. The full scan and fragment spectra were collected with resolution of 70,000 and 17,500, respectively. The source parameters are as follows: spray voltage: 3000 v; capillary temperature: 320 °C; heater temperature: 300 °C; sheath gas flow rate: 35; auxiliary gas flow rate: 10. Data analysis and quantitation were performed by the software Xcalibur 3.0.63 (Thermo Fisher, CA).

## Data Availability

All the data and materials are available by reasonable request.

## Abbreviations

*C. reinhardtii*: *Chlamydomonas reinhardtii*
TAG: Triacylglycerol
N: Nitrogen
TCA cycle: Tricarboxylic acid cycle
FDA: Fluorescein diacetate
CV: Contractile vacuole
G6P: Glucose-6-phosphate
F6P: Fructose-6-phosphate
F16BP: Fructose-1,6-diphosphate
GA3P: Glyceraldehyde-3-phosphate
3-PGA: 3-Phosphoglyceric acid
DHAP: Dihydroxyacetone phosphate
PEP: Phosphoenolpyruvic acid
G3P: Glycerol phosphate
LysoPA: Lysophosphatide
PA: Phosphatidic acid
DAG: Diacylglycerol
TAG: Triacylglycerol
AKG: Alpha-ketoglutarate
